# Evaluation of Elexacafor/Tezacaftor/Ivacaftor therapy after lung transplantation in Cystic Fibrosis: The Dutch National KOALA study

**DOI:** 10.1016/j.jhlto.2025.100210

**Published:** 2025-01-17

**Authors:** On behalf of the KOALA study group, Johanna P. van Gemert, Bart Luijk, Merel E. Hellemons, Klara A. Visser, Carina.M.E. Hansen, Renske van der Meer, C. Tji Gan, Hester van der Vaart, Onno W. Akkerman, Willie N. Steenhuis, Marieke Verkleij, Harry G.M. Heijerman, Erik A.M. Verschuuren

**Affiliations:** a Department of Pulmonary Diseases and Tuberculosis, University of Groningen, University Medical Center Groningen, Groningen, the Netherlands; b Department of Respiratory Medicine, University Medical Center Utrecht, Utrecht, the Netherlands; c Department of Respiratory Diseases, Erasmus MC Transplant Institute, Erasmus University, University Medical Center, Rotterdam, the Netherlands; d Department of Clinical Pharmacy & Pharmacology, University of Groningen, University Medical Center Groningen, Groningen, the Netherlands; e Department of Pulmonology, Haga Teaching Hospital, The Hague, the Netherlands; f Amsterdam UMC location University of Amsterdam, Emma Children’s Hospital, Child and Adolescent Psychiatry & Psychosocial Care, Amsterdam, the Netherlands

**Keywords:** CF, Lung transplant, ETI

## Abstract

**Background:**

Elexacaftor/Tezacaftor/Ivacaftor (ETI) for people with CF (PwCF) after lung transplantation (LTx) has been restrained due to uncertainties regarding efficacy and drug interactions. Given the persistence of extrapulmonary symptoms post-LTx, this prospective study aims to investigate the benefits and safety of ETI for PwCF post-LTx.

**Methods:**

Between Nov 2022-Nov 2023 ETI was offered to PwCF post-LTx with at least one F508del mutation in 3 Dutch LTx centers. PwCF were considered eligible if they had either a BMI ≤ 19 kg/m², chronic rhinosinusitis (CRS), uncontrolled diabetes or gastrointestinal (GI) symptoms. BMI, HbA1c, SNOT-22 score, GI Symptom Tracker, CF Questionnaire-Revised (CFQ-R), FEV_1_, creatinine, changes in calcineurin inhibitor (CNI) doses and levels were compared between baseline and 3 months follow-up.

**Results:**

Fifty-five PwCF post-LTx were included, of whom 5 were excluded because of ETI discontinuation due to side effects, within 3 month follow-up. Three months results showed a decrease in SNOT-22 score (*p*< 0.001) and GI symptoms (all 4, *p*< 0.05), an increase in BMI (*p*= 0.012) and CFQ-R (6 domains, *p*< 0.05). Median CNI daily dose had to be reduced from 6 to 4 mg (*p*< 0.001), to maintain stable CNI trough levels. Creatinine increased from 110 (87−141) to 115 (92−125) umol/L (*p*= 0.002).

**Conclusion:**

ETI for PwCF post-LTx shows favorable effects on CRS, GI symptoms, and quality of life, but not on BMI and HbA1c. Due to its high cost, careful consideration and further studies are required. Monitoring renal function and CNI trough levels is recommended.

## Introduction

Cystic fibrosis (CF) arises from mutations in the cystic fibrosis transmembrane conductance regulator (CFTR) gene.^1^ CF is a life-threatening disease that was previously a common indication for lung transplantation (LTx). The CFTR modulator Elexacaftor/Tezacaftor/Ivacaftor (ETI) shows markedly enhanced pulmonary function and reduced exacerbation frequency in people with CF (PwCF).^1^, ^2^, ^3^ Consequently, this improvement in pulmonary function and number of exacerbations reduced the LTx indication in PwCF.^4^ Historically, PwCF post-LTx were not considered eligible for ETI treatment due to uncertainty regarding direct benefits and potential drug-drug interactions with immunosuppressive therapy, primarily calcineurin inhibitors (CNI). However, CF affects multiple organ systems, leading to extrapulmonary symptoms post-LTx that may impact quality of life and graft function post-LTx as well.

While most research on ETI has primarily focused on pulmonary functions, earlier studies in PwCF without LTx show improvements in extrapulmonary conditions including Body Mass Index (BMI), chronic rhinosinusitis (CRS), CF-related diabetes (CFRD), pancreatic insufficiency, and CF-associated liver disease.^5^ Importantly, Ramos et al. demonstrated in a retrospective cohort study in PwCF post-LTx that ETI improved HbA1c, increased hemoglobin levels in those with anemia, and reduced antibiotic prescription frequency. However, a substantial proportion of PwCF (42%) discontinued ETI therapy at a median of 56 days.^6^ Therefore, to further explore the benefit of ETI post-LTX, it is necessary to conduct further prospective studies on the benefits and to evaluate drug-drug interactions and side effects.

The primary aim of the prospective Dutch multicenter KOALA study is to investigate the potential benefits on BMI, gastro intestinal (GI) complaints, DM regulation, CRS, pulmonary function and quality of life of ETI for PwCF post-LTx. Secondary objectives include assessing adverse events (AE), the effect of ETI on CNI trough levels and determining the required CNI dose adjustments to maintain stable trough levels. In this manuscript, we will present the three-month results.

## Material and methods

### Study patients

All Dutch PwCF post-LTx were eligible for inclusion if they had at least one F508del mutation in the CFTR gene and one of the following: underweight (BMI ≤19 kg/m² and/or enteral nutrition), CRS (CF-related CRS management, history of surgery), poor control of CFRD (>50EH insulin/day and/or HbA1C > 60 mmol/mol) or GI manifestations (history of distal intestinal obstruction syndrome (DIOS), use of laxatives). Patients were excluded if they had issues with medication adherence or if they had severe liver cirrhosis (Child-Pugh C), confirmed by liver ultrasound.

### CF care

In the Netherlands, there are 7 CF centers and 3 transplantation centers. All 3 transplantation centers also provide CF care. Post-transplant care is conducted collaboratively by both the transplantation physicians and the CF specialists. This collaboration is characterized by thorough communication and close coordination between healthcare providers. ETI may be prescribed in the Netherlands to all PwCF with one F508del mutation and is not restricted to study settings.

### Transplant care

Follow-up of all LTx patients took place every 3 months for monitoring of transplant function as part of standard care with lab results and pulmonary function tests (PFT). All patients received maintenance immunosuppression: tacrolimus, prednisolone and mycophenolate mofetil (MMF). Alternative immunosuppressants were cyclosporine, azathioprine, everolimus or sirolimus. Post-transplantation prophylactic therapy included: cotrimoxazole for Pneumocystis pneumonia and acyclovir for Herpes virussus and valganciclovir depending on Cytomegalovirus (mis)match.

### Study medication

PwCF started with the full ETI dosage: in the morning, 2 tablets containing ivacaftor 75 mg/tezacaftor 50 mg/elexacaftor 100 mg, and in the evening, 1 tablet of 150 mg ivacaftor. However, in patients with liver cirrhosis, confirmed by liver ultrasound, or those who have undergone a liver transplant, the dosage was adjusted to alternate between 2 tablets of ivacaftor 75 mg/tezacaftor 50 mg/elexacaftor 100 mg one day and 1 tablet of ivacaftor 75 mg/tezacaftor 50 mg/elexacaftor 100 mg the next day. The additional evening dose of ivacaftor was also omitted. When used concurrently with azoles, the dosage was adjusted to 2 tablets of ivacaftor 75 mg/tezacaftor 50 mg/elexacaftor 100 mg twice a week, with no additional ivacaftor in the evening. The above dosage recommendations were made based on the product information for ETI.^7^ All patients were instructed to take their ETI with fat-containing food.

### Study design

The KOALA study is a Dutch national prospective multicenter study. The study was granted a waiver by the respective Ethical Committees, as PwCF post-LTx were to receive ETI as part of their standard care. The study was conducted exclusively in the 3 transplantation centers and not in the CF centers that are not transplantation centers. All participants provided written informed consent prior to enrollment in the study. The study complies with the International Society for Heart and Lung Transplantation (ISHLT) Ethics Statement.

PwCF were recruited at one of the 3 Dutch LTx centers, where their follow-up also took place. Regular online meetings were held between the centers, and the study data were anonymously entered into a shared RedCap database. The principal investigator, based at one of the centers, compiled the data, conducted the analyses, and coordinated the study. A study agreement was signed by all 3 participating LTx centers.

In all 3 centers, a multidisciplinary team meeting was held to review which PwCF were eligible to enroll in the study according to the inclusion criteria as mentioned above. These PwCF were invited to participate in the study and were sent informational materials. Patients could be included between November 1, 2022, and November 1, 2023. Before starting ETI, PwCF were seen at the outpatient clinic during their standard LTx follow-up appointment by the transplant- or CF physician. During this visit, they received an explanation about the ETI, possible AE of the ETI, and the study visits. If they agreed to participate, baseline parameters were collected and a sweat test was conducted. Baseline parameters were gathered as part of standard care for LTx: BMI, blood tests (HbA1c, kidney function, liver enzymes, CNI trough levels) and PFT. Spirometry was performed according to ATS/ERS guidelines.^8^ Additionally, three questionnaires to asses extrapulmonary parameters were utilized, including the Sino-Nasal Outcome Test (SNOT-22) for CRS, the GI Symptom Tracker for GI symptoms and the CF Questionnaire-Revised (CFQ-R) for quality of life. Follow-up visits, with lab result, PFT and the questionary’s took place 3 months after starting ETI. The sweat test was only repeated after three months. For safety reasons, kidney function, liver enzymes, and CNI trough levels were measured at 2, 4, and 8 weeks after start of ETI and were discussed with the patient over the phone or at the clinic. Dose adjustments of the CNI were documented at every visit.

AE were assessed at every study visit. Patients were questioned about the potential occurrence of suspected AE of special interest including GI symptoms, headache, itching, upper airway infections, psychological side effects, decrease in FEV1 at home monitoring, weight and ear, nose and throat (ENT) symptoms.

### Questionnaires

The CFQ-R is a patient-reported multiple-domain questionnaire specific for PwCF, to measure health-related quality of life. The score consists of 9 quality of life domains (physical functioning, role functioning, vitality, emotional functioning, social functioning, body image, eating disturbances, treatment burden, health perception) and 3 symptom scales (weight, respiratory, and digestion). Responses of the 12 items are standardized from 0 to 100, with higher scores indicating better quality of life.^9^ The SNOT-22 is a patient-reported measure of outcome developed for use in CRS with or without nasal polyposis. The SNOT contains 22 individual questions, with higher score indication more severe disease (supplement 1).^10^

The Dutch GI Symptom Tracker (supplement 2) is a standardized measure of GI symptoms, enzyme and nutrition adherence, demonstrating good reliability and validity.^11^, ^12^, ^13^ It consists of 4 domains: Eating Challenges (4 items), Stools (8 items), Adherence Challenges (5 items), and Abdominal Symptoms (7 items). Scores are standardized on a 0-to-100 scale with higher scores indicating more symptoms.

### Study end-points

Previous studies on ETI in PwCF used lung function as the primary outcome. Since this is not a suitable outcome measure for LTx patients, we selected extrapulmonary manifestations as the primary outcome measure. Consequently, the primary outcome consists of several parameters, namely: BMI, HbA1c, SNOT-22 score, GI Symptom Tracker score, CFQR score and FEV1 pre- and 3 months post-ETI. Secondary outcomes were, the effect of ETI on CNI trough levels, kidney function and liver enzymes, the CNI dose adjustment needed to maintain stable trough levels and AE. Primary and secondary outcomes were measured at each visit as described in the study calendar (supplement 3).

### Statistical analysis

Analyses were carried out using IBM SPSS for Mac, version 24.0. Continuous variables are expressed as median (interquartile range). Wilcoxon signed-rank test were used for within-group analyses, to compare the study parameters before initiation of ETI and at 3 months follow-up. A *p*-value of less than 0.05 is used as the cut-off for significance. The CNI through levels were compared between baseline and 2, 4, 8 and 12 weeks follow-up.

## Three-month results

### Patients

Between November 2022 and November 2023, 55 PwCF post-LTx were included in the study in the 3 Dutch transplant centers. The flowchart detailing the inclusion and exclusion criteria is provided in Supplement 4. Of those, 7 PwCF received an adjusted dosage due to liver cirrhosis (n = 5) or liver transplantation (n = 2), and 1 received an adjusted dosage due to concurrent use of azoles. Five PwCF (9%) were excluded due to discontinuation of ETI, because of AE before the 3-month follow-up. These AE were: psychological AE (n = 2), headache (n = 1), lung function decline (FEV1) (n = 1), muscle cramps in legs (n =1). Consequently, 3-month follow-up parameters were available for 50 PwCF. Baseline characteristics are presented in Table 1. The median age was 42 years (IQR 34–49), and 31/50 PwCF (62%) were male. Bilateral transplant was performed in all of the PwCF. The median time post-LTx was 11 years (IQR 7–15 years). More than half (34/50; 68%) of the PwCF were homozygous for the F508del mutation. The median BMI was 22 kg/m² (IQR 22–24). Additionally, 41/50 ( 82%) of the PwCF had both endocrine and exocrine pancreatic insufficiency. GI symptoms were present in 30/50 (60%) of the PwCF, cirrhosis (Child-Pugh A or B) in 7/50 (14%), and CRS in 40/50 (80%). CLAD was present in 12/50 (24%) of the PwCF. The median sweat chloride level at baseline was 117 mmol/L (IQR 106–138 mmol/L). Of the PwCF post-LTx 48/50 (98%) used CNI as part op their immunosuppressive therapy post-LTx.

**Table 1:** Baseline Characteristics of the 50 People with Cystic Fibrosis after Lung Transplantation

Variable	
Age, years	42 (34−49)
Time after LTx, years	11 (7−15)
Gender, male (%)	31 (62)
F508del mutation 1 copy, n (%)	50 (100)
F508del mutation 2 copies, n (%)	34 (68)
Bilateral LTx, n (%)	50 (100)
BMI, kg/m^2^	22 (22−24)
Pancreas insufficiency	
Exocrine only, n (%)	8 (16)
Endocrine and exocrine, n (%)	41 (82)
GI symptoms*, n (%)	30 (60)
CF related cirrhosis, n (%)	7 (14)
CRS, n (%)	40 (80)
CLAD, n (%)	12 (24)
Sweat chloride, mmol/L	117 (106−138)

Continuous variables are expressed as median (interquartile range)

LTx, lungtransplantation; BMI, Body Mass Inex, GI, gastrointestinal; CF, cystic fibrosis, CRS, chronic rhinosinusitis, CLAD, chronic lung allograft dysfunction

* GI symptoms: history of distal intestinal obstruction syndrome (DIOS) and/or use of laxatives

### Primary outcomes

The 3-month results are shown in Table 2. Sweat chloride significantly decreased from 117 mmol/L (IQR 106–138) before the start of ETI to 66 mmol/L (IQR 48–75) after 3 months of ETI treatment (*p* < 0.001). There was a significant, but no clinically relevant, increase in BMI from 21.7 kg/m^2^ (19.8–23.6) to 21.8 kg/m^2^ (19.7–24.1) (*p* = 0.012). In 11 patients with a BMI less than 19, there was no significant rise in BMI after 3 months of ETI (before ETI 16.9 21.7 kg/m^2^, after 3 months 17.8 21.7 kg/m^2^; *p* = 0.208).

**Table 2:** Parameters at Baseline and 3 Months after Starting Elexacaftor/Tezacaftor/Ivacaftor

Parameter	Baseline	3 months	*p*-value
Sweat Chloride, mmol/L	117 (106−138)	66 (48−75)	< 0.001
BMI, kg/m^2^	21.7 (19.8−23.6)	21.8 (19.7−24.1)	0.012
HbA1c, %	6.8 (6.1−7.5)	7.0 (6.2−7.7)	0.312
SNOT−22 sum score	31.5 (16.8−49.0)	12.5 (5.3−19.8)	<0.001
GI Symptom Tracker score			
Abdominal symptoms	48 (38−61)	36 (32−43)	<0.001
Stools	58 (47−66)	47 (37−54)	<0.001
Eating challenges	38 (33−50)	31 (25−38)	0.002
Adherence challenges	32 (25−40)	25 (25−35)	0.011
FEV1, L	2.7 (2.1−3.3)	2.7 (2.1−3.2)	0.076
Creatinine, µmol/L	110 (87−141)	115 (92−145)	0.002
EGFR, ml/min/1,73 m2	68 (48−85)	63 (43−79)	0.010
Liver enzymes			
AST, U/L	18 (13−28)	20 (14−30)	0.388
ALT, U/L	23 (15−35)	24 (18−31)	0.485
ALP, U/L	89 (67−115)	82 (67−112)	0.023
GGT, U/L	26 (17−51)	25 (18−43)	0.436
LDH, U/L	205 (183−230)	221 (193−239)	0.051
CNI trough level, µg/ml	7.0 (6.3−8.6)	7.6 (6.1−9.1)	0.077
CNI dose, mg/day	6.0 (4.0−7.1)	4.0 (3.0−7.0)	<0.001

Continuous variables are expressed as median (interquartile range)

BMI, Body Mass Index; SNOT, Sino-Nasal Outcome Test; GI, gastrointestinal; FEV1, Forced Expiratory Volume; EGFR, estimated glomerular filtration rate; AST, aspartate aminotransferase; ALT, Alanine transaminase; ALP, Alkaline phosphatase, GGT, Gamma-glutamyltransferase; LDH, Lactate dehydrogenase; CNI, calcineurin inhibitor

**Figure 1:**
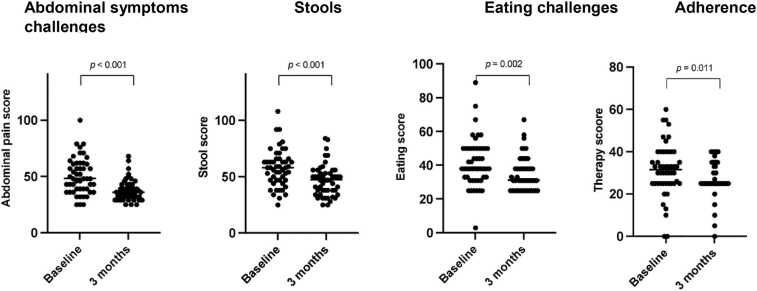
Results of the Gastrointestinal Symptom Tracker at baseline and 3 months follow-up. Gastrointestinal symptom tracker results were available for all 50 patients at baseline and 3 months follow-up.

**Figure 2:**
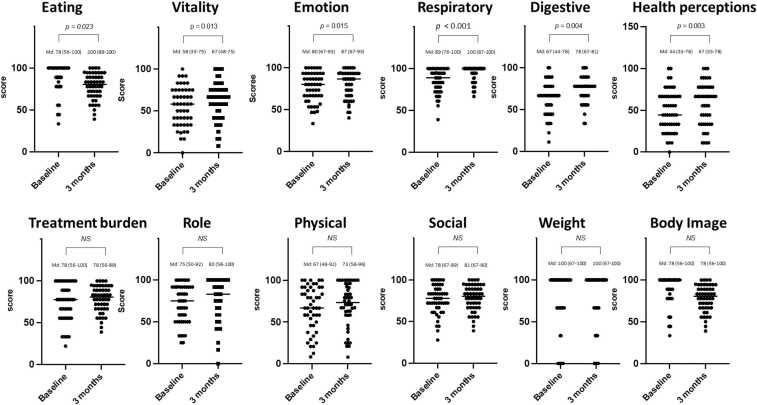
Results of the CF Questionnaire-Revised at baseline and 3 months follow-up. CF Questionnaire-Revised results were available for 49 patients at baseline and for 50 patients 3 months follow-up. NS, not significant; Md, median with interquartile range.

HbA1c remained unchanged and insulin usage did not significantly decrease after ETI treatment. Similarly, in the group with diabetes, HbA1c did not change significantly after 3 months of ETI. There was a significant decrease in SNOT-22 score from 31.5 (IQR 18.8–49) at baseline to 12.5 (IQR 5.3–19.8) at 3 months follow-up (*p* < 0.001). The percentage of PwCF who achieved the Minimal clinically important differences (MCID) SNOT-22 score threshold of 8 was 69%.^14^ There was a significant improvement in all 4 domains of the GI symptom tracker (Table 2 and Figure 1). Six out of the 12 domains of the CFQ-R significantly improved (Figure 2). The domains that showed improvement were: eating disturbances, vitality, emotional functioning, respiratory symptoms, digestive symptoms and health perceptions. FEV1 remained unchanged, 2.7 L (IQR 2.1–3.3) at baseline and 2.7 L (IQR 2.1–3.2) at 3 months of ETI treatment.

### Pharmacology

**Figure 3:**
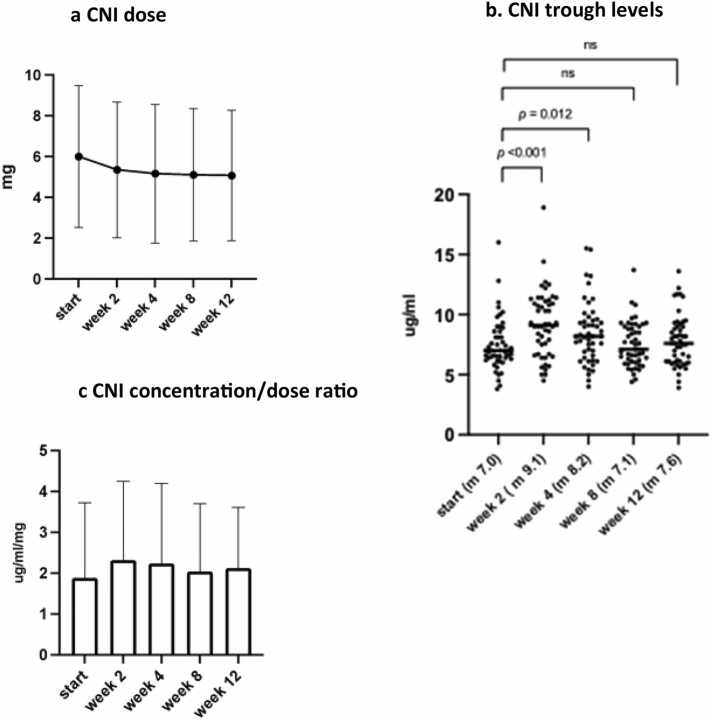
**Calcineurin inhibitor trough levels dosage and concentration/dose concentration at baseline and 3 months follow-up.** CNI, calcineurin inhibitor; ns, not significant; m, median.

CNI trough levels rose directly after the start of the ETI from 7.0 to 9.1 μg/L but stabilized after dose adjustment to 7.6 μg/L (Figure 3). CNI dose decreased from 6 mg/day before start of ETI to 4 mg/day after 3 months of ETI treatment. Therefore, ETI necessitated a dose reduction of 33% to maintain stable CNI through levels. The CNI concentration/dose ratio significantly increased from 1.16 μg/L* 1/mg to 1.75 μg/L* 1/mg after 3 months of ETI treatment (Figure 3).

### Adverse events

**Figure 4:**
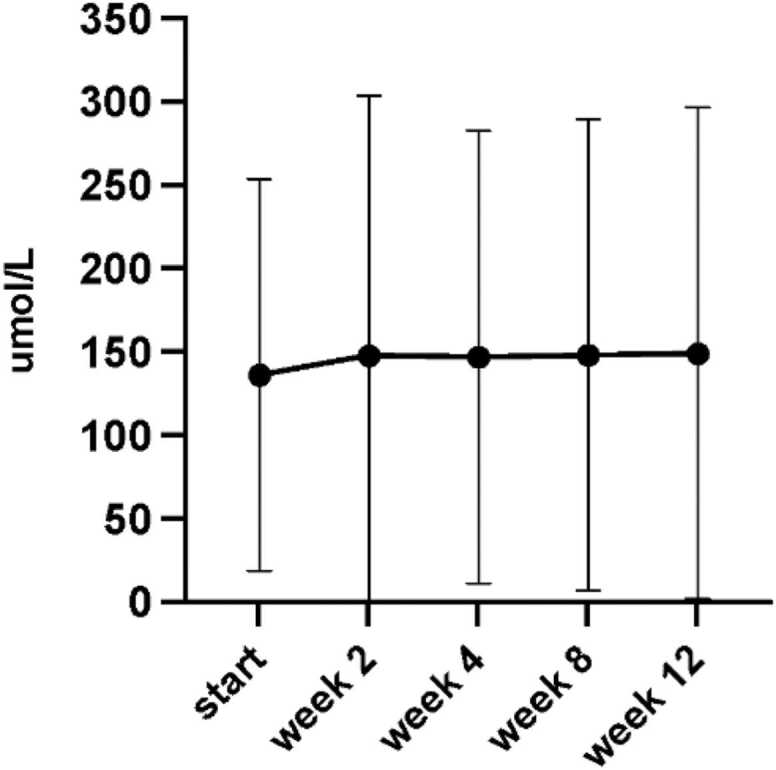
Creatinine at baseline and 2,4, 8 and 12 week follow-up.

There was no effect of ETI on liver enzymes, except for ALP which declined from 89 U/L (IQR 76–115) to 82 U/L (IQR 67–112). Creatinine increased after the start of ETI but stabilized after CNI dose adjustment, although remained higher than at baseline (Table 2, Figure 4).

As a result of ETI, 46 AE occurred in 22/50 (46%) PwCF. The most common AE were GI symptoms, headache, upper respiratory infections, pruritus, and liver enzyme abnormalities. In 77% of cases, these adverse events resolved spontaneously after approximately 2 weeks (Table 3).

**Table 3:** Side Effects of Elexacaftor/Tezacafor/Ivacaftor in People with Cystic Fibrosis after Lung Transplantation

	ENT	GI	UAI	Liver	Headache	LF Decline	Rash	Psychiatric	Fever	Weight Gain
2 weeks	5	10	1	4	7	0	6	1	1	0
4 weeks	2	3	2	1	0	2	1	1	0	0
8 weeks	1	4	0	1	2	0	1	3	0	0
12 weeks	0	2	2	1	1	0	1	3	0	1

ENT, ear/nose/throat; GI, gastrointestinal; UAI, upper airway infections; LF, lung function

There was 1 hospitalization due to a viral upper airway infection and no deaths related to ETI during the study period.

## Discussion

The KOALA study is the first prospective multicenter study examining the effect of ETI in PwCF post-LTx. Our findings indicate that ETI has favorable effects on CRS, GI symptoms, and overall quality of life. These results are consistent with 3 small retrospective studies (total n = 27), which all demonstrated improvements in weight, CRS, and GI symptoms following ETI treatment in PwCF.^15^, ^16^, ^17^ However, Ramos et al. reported, in a larger study cohort in 94 PwCF post-LTx, no effect of ETI on BMI, but did find improvements on HbA1c levels, increased Hemoglobin levels in those with anemia, and a reduced frequency of antibiotic prescriptions.^18^

Contrary to Ramos et al., our study did not observe significant effects on HbA1c. Additionally, insulin usage among PwCF in our study did not significantly decrease after ETI treatment. The lack of an effect on HbA1c may also be due to the fact that the post-transplant immunosuppressive use of steroids and tacrolimus can also induce diabetes.

Remarkably, almost 42% of the PwCF discontinued ETI in the study of Ramos et al.^18^ In our study, only 5/55 (9%) PwCF discontinued ETI before the 3-month follow-up period. Many of our PwCF (46%) experienced AE, but our findings showed that these typically resolve spontaneously after a few weeks. As a result, it is crucial to provide PwCF with adequate information regarding this matter with our gained knowledge about AE in PwCF without transplantation. Another important thing to realize is that PwCF post-LTx do not anticipate the same benefits of ETI as non-transplanted PwCF, such as prolonged life expectancy. Consequently, they might be less willing to tolerate ETI related AE. Additionally, PwCF post-LTx are highly protective of their transplanted lungs and may fear that ETI could harm their transplanted lungs. Fortunately, our study demonstrated that ETI has no detrimental effect on pulmonary function, as previously observed in the study by Benninger et al.^16^

Given the high costs of ETI, it is important to weigh whether the benefits outweigh these costs. CF is a heterogeneous multi-organ disease, in which patients after LTx may still suffer from many debilitating conditions such as DIOS, CRS, uncontrolled diabetes, pancreatitis, and more. The burden of care for PwCF post-LTx is high and includes regular local care of sinuses, routine clinical and diagnostic check-ups. CRS can result in local and systemic complications, sometimes associated with hospitalization for prolonged intravenous antibiotic treatment of the upper and lower airways and airway colonization with pathogens such as *Pseudomonas*.^19^ Frequent infections, especially *Pseudomonas* infections, may lead to CLAD.^20^ However, we do not yet know whether ETI can also prevent CLAD. We will have to wait for the long-term results of the KOALA study to determine if ETI has an impact on CLAD development. Although the results of ETI appear promising for questionnaire-based endpoints, unfortunately, there is no effect on measurable values such as BMI and HbA1c. Given the high cost of ETI, a careful consideration is required, and further studies with cost-benefit analyses and more concrete measurable endpoints are needed to strike the balance. In addition, it is important to determine which PwCF post-LTx benefit the most from ETI. In the study by Ramos et al., prescription practice patterns varied across LTx programs, reflecting variability at the provider level.^18^ The inclusion criteria we used to determine whether or not to administer ETI were clear to all centers and prescribers. Future study results hopefully give answer to which patients benefit the most from ETI.

In our study, we showed that ETI necessitated a CNI dose reduction of 33% to maintain stable trough levels. This is in line with the study by Dolaginsky et al., which demonstrated a required dose reduction of 50% and with van der Meer et al., who showed that five dose adjustments were performed in 4 liver transplant patients in order to attain CNI target range.^15^, ^21^ In contrast, Benninger et al. showed no significant impact on the immunosuppressive drug doses.^16^ The dose reduction needed can be explained by several reasons. First, both ETI and CNI are substrates of permeability glycoprotein (P-gp). P-gp is an efflux transporter present on the apical side of the intestinal epithelium that prevents intracellular accumulation of CNI and ETI by increasing their efflux to the intestinal lumen.^22^ In theory, if more than one substrate competes to bind with P-gp, it decreases the likelihood of the drugs being pumped out of the cell, resulting in accumulation and higher trough levels. The second reason might be that the effect of ETI on the GI tract increases the Gl absorption of CNI. With the standard dosage of ETI, the variability between PwCF in exposure as measured by AUC or Cmin is high.^7^ Third, metabolization of ETI can be influenced by medication that are CYP3A4 liver enzyme inductors or inhibitors such as azoles. The latter can influence treatment start decision and increases side effects. To better understand the relationship between ETI exposure and the level of drug-drug interaction with CNI we are planning to determine trough levels of ETI in all PwCF included in our study.

Notably, in our study, renal function declined immediately after the initiation of ETI. Although this stabilized after reducing the CNI dose, it did not return to baseline. Since ETI is minimally cleared renally, the decline is most likely due to rising CNI levels immediately after starting ETI. It is possible that this decline in renal function can be prevented by adjusting the CNI dose by approximately 30% at the start of ETI. Additionally, the long-term effects of ETI on renal function should be examined over a longer follow-up period.

There are some limitations to our study that should be considered when interpreting our findings. This was a nationwide prospective observational cohort study. The small sample size, and the lack of a comparison group limits the reliability of the results. Unfortunately, the low prevalence of PwCF post-LTx constrains patient studies and data collection, which is a common reality in LTx research. Secondly, not all patients started with a full dose of ETI, potentially leading to an underestimation of ETI's effects. Third, our study showed a favorable safety profile on the short term but longer term safety profile needs to be elucidated. Fourth, the median time between LTx and start of the ETI was 11 years in our study. Therefore, our results might not be applicable to LTx patients shortly after LTx who start ETI. Fifth, we used validated questionnaires that are inherently subjective and may not be suitable for the post-transplant group of PwCF. Furthermore, the MCID is only established for the respiratory domain of the CFQR, while it remains unavailable for the other domains.^23^ As a result, we were unable to apply the MCIDs, although they are crucial for enhanced clinical interpretation of the results. Also, for sinus disease evaluation we did not perform a CT scan of the sinuses because it would have subjected patients to additional tests and radiation, which did not align with our non-WMO application. Finally, some patients may have taken their ETI together with tacrolimus and fat-containing food, while others may have taken tacrolimus at a different time than the ETI. This could potentially result in differences in drug absorption between patients.

## Conclusion

This is the first prospective multicenter study to investigate the effect of ETI in PwCF post-LTx. We showed that ETI post-LTx has favorable effects on CRS, GI symptoms and quality of life. However, careful monitoring of renal function and CNI levels are recommended.
