# Supplementary material for: Evaluation of Elexacafor/Tezacaftor/Ivacaftor therapy after lung transplantation in Cystic Fibrosis: The Dutch National KOALA study
Source: JHLT Open. 2025 Jan 17;7:100210. doi: 10.1016/j.jhlto.2025.100210 (PMC11935345; doi:10.1016/j.jhlto.2025.100210)
Supplement: Supplementary file 1 — Supplemental material [file mmc1.pdf]

Neus-keel-oorziekten - Hoofd- Halschirurgie
Dr.Carl Van Laer - Dr. Jorn Potvin
www.nkokleinbrabant.be

Neusklachten Vragenlijst (Sino-Nasal Outcome Test-22 Questionnaire)

Hieronder vindt u een lijst met symptomen en sociale/emotionele gevolgen van de aandoening van uw neus. Gelieve volgende vragen zo goed mogelijk te beantwoorden. Er zijn geen juiste of foute antwoorden, en alleen u kan ons deze informatie geven. Kan u een score geven aan uw problemen zoals u ze de laatste tijd ondervond?

Geef een nummer aan elk van de onderstaande klachten. →	**Geen probleem**	**Zeer licht probleem**	**Licht of weinig probleem**	**Matig probleem**	**Ernstig probleem**	**Kan niet slechter**
1. Nood tot het snuiten van de neus	0	1	2	3	4	5
2. Niezen	0	1	2	3	4	5
3. Loopneus/neusloop	0	1	2	3	4	5
4. Hoest	0	1	2	3	4	5
5. Postnasale drip (neusloop achteraan in de keel))	0	1	2	3	4	5
6. Taaie neusloop	0	1	2	3	4	5
7. Volheidsgevoel in oren	0	1	2	3	4	5
8. Duizeligheid	0	1	2	3	4	5
9. Oorpijn/druk in het oor	0	1	2	3	4	5
10. Gelaatspijn/druk	0	1	2	3	4	5
11. Moeilijkheid in slaap vallen	0	1	2	3	4	5
12. 's nachts wakker worden	0	1	2	3	4	5
13. Gebrek aan goede nachtrust	0	1	2	3	4	5
14. Vermoeid wakker worden	0	1	2	3	4	5
15. Vermoeidheid overdag	0	1	2	3	4	5
16. Verminderde productiviteit	0	1	2	3	4	5
17. Verminderde concentratie	0	1	2	3	4	5
18. Frustratie/rusteloos/prikkelbaar	0	1	2	3	4	5
19. Neerslachtig	0	1	2	3	4	5
20. Beschaamdheid	0	1	2	3	4	5
21. Smaakzin/reukzin	0	1	2	3	4	5
22. Verstopte neus	0	1	2	3	4	5

SUBTOTAAL: ______ ______ ______ ______ ______

TOTAAL: ______
