# Supplementary material for: Evaluation of Elexacafor/Tezacaftor/Ivacaftor therapy after lung transplantation in Cystic Fibrosis: The Dutch National KOALA study
Source: JHLT Open. 2025 Jan 17;7:100210. doi: 10.1016/j.jhlto.2025.100210 (PMC11935345; doi:10.1016/j.jhlto.2025.100210)
Supplement: Supplementary file 2 — Supplemental material [file mmc2.pdf]

# GI Symptom Tracker

This gastrointestinal (GI) symptom tracker is designed to help you understand the impact of GI symptoms and treatments on your everyday life. Share the information with your healthcare team. Thank you for your willingness to answer these questions.

*These questions are about your current health, as you perceive it. There are no right or wrong answers!*
*If you are not sure how to answer, choose the response that seems closest to your situation. Please share these results with your CF care team.*

A. Please indicate the strength of the enzyme you are currently taking: _________

How many enzymes does your health care provider tell you to take with:
B. Each meal ______ C. Each snack ______

During the past week, indicate how often:

| | Almost Always | Often | Sometimes | Never |
|---|---|---|---|---|
| 1. Your appetite was poor | ❍ | ❍ | ❍ | ❍ |
| 2. You had to rush to the bathroom because of GI problems | ❍ | ❍ | ❍ | ❍ |
| 3. You felt bloated | ❍ | ❍ | ❍ | ❍ |
| 4. You had to stay on the toilet for a long time | ❍ | ❍ | ❍ | ❍ |
| 5. You had acid reflux (heartburn) | ❍ | ❍ | ❍ | ❍ |
| 6. You skipped a meal | ❍ | ❍ | ❍ | ❍ |
| 7. You forgot to take your enzymes | ❍ | ❍ | ❍ | ❍ |

During the past week, how *bothered* were you by:

| | A Great Deal | Somewhat | A Little | Not At All |
|---|---|---|---|---|
| 8. Fatty or greasy foods | ❍ | ❍ | ❍ | ❍ |
| 9. Taking enzymes in front of others | ❍ | ❍ | ❍ | ❍ |
| 10. Constipation | ❍ | ❍ | ❍ | ❍ |
| 11. Stomach ache | ❍ | ❍ | ❍ | ❍ |
| 12. Cramping | ❍ | ❍ | ❍ | ❍ |

How often in the past week have you had:

| | Almost Always | Often | Sometimes | Never |
|---|---|---|---|---|
| 13. Gas | ❍ | ❍ | ❍ | ❍ |
| 14. Loose stools | ❍ | ❍ | ❍ | ❍ |
| 15. Bad-smelling stools | ❍ | ❍ | ❍ | ❍ |
| 16. Greasy stools | ❍ | ❍ | ❍ | ❍ |
| 17. Diarrhea | ❍ | ❍ | ❍ | ❍ |

During the past week:
18. How many stools did you have per day? (Please provide number or range): ________
During the past week, indicate how often:

| | Almost Always | Often | Sometimes | Never |
|---|---|---|---|---|
| 19. You were embarrassed about using a public bathroom | ❍ | ❍ | ❍ | ❍ |

| | Almost Always | Often | Sometimes | Never | Does not apply |
|---|---|---|---|---|---|
| 20. You forgot to take medications to help your digestive system (e.g., antacids, laxatives) | ❍ | ❍ | ❍ | ❍ | ❍ |

| | Almost Always | Often | Sometimes | Never |
|---|---|---|---|---|
| 21. You had enough time to eat | ❍ | ❍ | ❍ | ❍ |
| 22. You missed school, work, or daily activities because of GI problems | ❍ | ❍ | ❍ | ❍ |
| 23. You forgot to bring your enzymes with you | ❍ | ❍ | ❍ | ❍ |
| 24. You relied on "fast food" or snacks like soda, chips, or candy to boost your calories | ❍ | ❍ | ❍ | ❍ |
| 25. You had GI problems (stomach ache, loose stools) because you may have missed your enzymes | ❍ | ❍ | ❍ | ❍ |

**The GI Symptom Tracker is intended for informational purposes only and should not be used as a substitute for advice provided by your doctor or other healthcare professionals.**

**© 2017 AbbVie Inc. and Dr. Alexandra Quittner. All rights reserved. No part of this material may be used or reproduced without the prior written permission of the copyright owners.**
