# Supplementary material for: Evaluation of Elexacafor/Tezacaftor/Ivacaftor therapy after lung transplantation in Cystic Fibrosis: The Dutch National KOALA study
Source: JHLT Open. 2025 Jan 17;7:100210. doi: 10.1016/j.jhlto.2025.100210 (PMC11935345; doi:10.1016/j.jhlto.2025.100210)
Supplement: Supplementary file 3 — Supplemental material [file mmc3.docx]

**Supplement 3. study calendar of the KOALA study**

|  | **Visit 1**  **Start** | **Visit 2**  **2 weeks** | **Visit 3**  **4 weeks** | **Visit 4**  **8 weeks** | **Visit 4**  **8 weeks** | **Visit 6**  **1/2 year** | **Visit 7**  **1 year** |
| --- | --- | --- | --- | --- | --- | --- | --- |
| **Weight** | X | X | X | X | X | X | X |
| **Lab results:**   - Creatinine - Liver enzymes - CNI trough level - HbA1c | X  X  X  X | X  X  X  X | X  X  X  X | X  X  X  X | X  X  X  X  X | X | X |
| **PFT** | X |  |  |  | X | X | X |
| **Sweat test** | X |  |  |  | X |  |  |
| **Side effects** |  | X | X | X | X | X | X |
| **Questionnaires**   - SNOT score - Quality of live - GI Symptom Tracker | X |  |  |  | X | X | X |
| CNI, calcineurin inhibitor; PFT, pulmonary function tests, SNOT, Sino-Nasal Outcome Test; GI, gastrointestinal | | | | | | | |
