# Supplementary figures and images for: Evaluation of Elexacafor/Tezacaftor/Ivacaftor therapy after lung transplantation in Cystic Fibrosis: The Dutch National KOALA study
Source: JHLT Open. 2025 Jan 17;7:100210. doi: 10.1016/j.jhlto.2025.100210 (PMC11935345; doi:10.1016/j.jhlto.2025.100210)

**Supplement 4. study flowchart of the KOALA study**


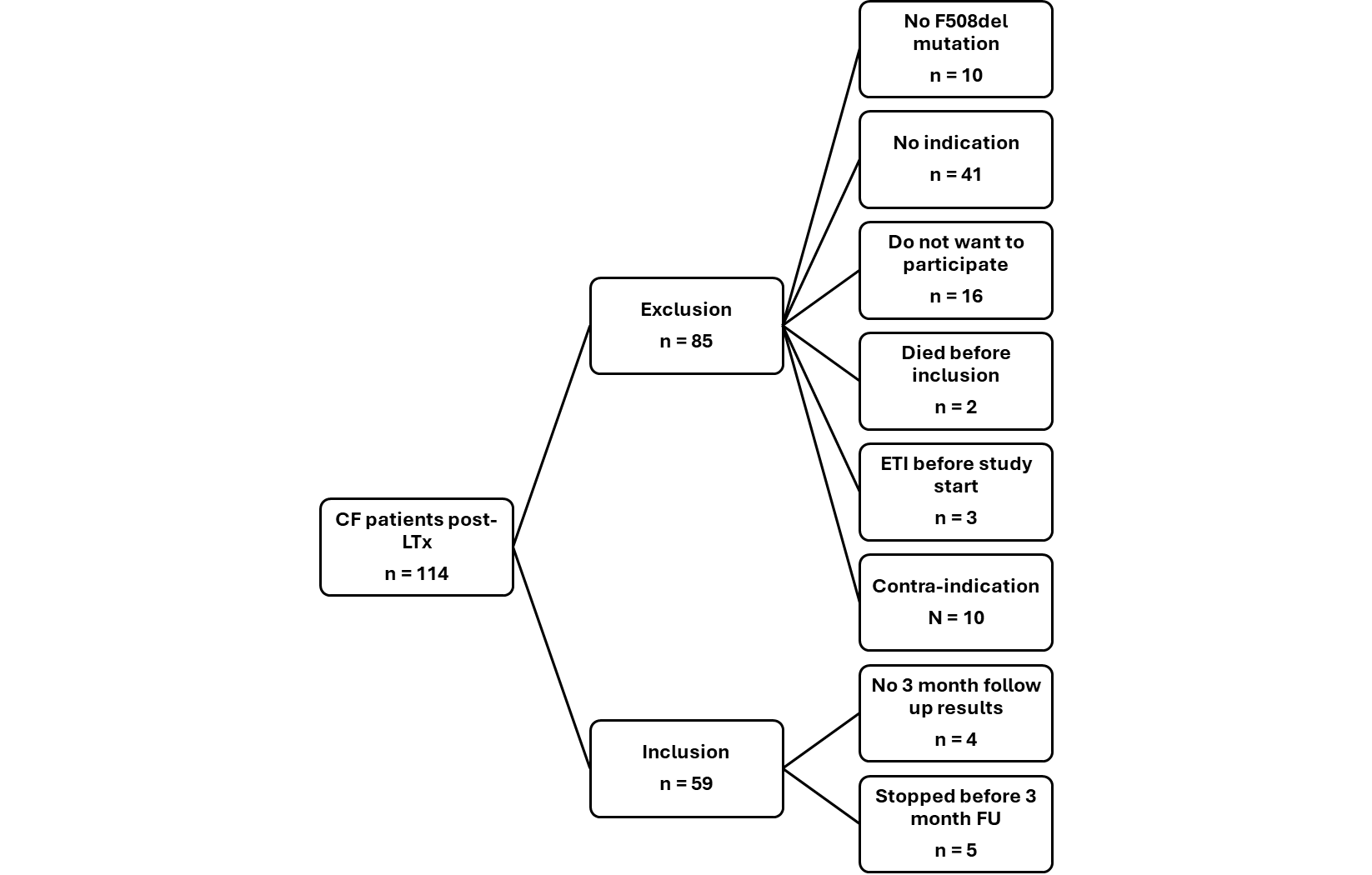

Supplement: Supplementary file 4 — Supplemental material [file mmc4.docx]
